# A chromosome-level genome assembly of the spider mite *Tetranychus piercei* McGregor

**DOI:** 10.1038/s41597-024-03189-0

**Published:** 2024-04-05

**Authors:** Lei Chen, Xin-Yue Yu, Feng Zhang, Hua-Meng Zhang, Li-Xue Guo, Lu Ren, Xiao-Yue Hong, Jing-Tao Sun

**Affiliations:** Department of Entomology, Nanjing Agricultural University, Nanjing, Jiangsu 210095 China

**Keywords:** Genomics, Genome

## Abstract

Despite the rapid advances in sequencing technology, limited genomic resources are currently available for phytophagous spider mites, which include many important agricultural pests. One of these pests is *Tetranychus piercei* (McGregor), a serious banana pest in East Asia exhibiting remarkable tolerance to high temperature. In this study, we assembled a high-quality genome of *T. piercei* using a combination of PacBio long reads and Illumina short reads sequencing. With the assistance of chromatin conformation capture technology, 99.9% of the contigs were anchored into three pseudochromosomes with a total size of 86.02 Mb. Repetitive elements, accounting for 14.16% of this genome (12.20 Mb), are predominantly composed of long-terminal repeats (30.7%). By combining evidence of *ab initio* prediction, transcripts, and homologous proteins, we annotated 11,881 protein-coding genes. Both the genome and proteins have high BUSCO completeness scores (>94%). This high-quality genome, along with reliable annotation, provides a valuable resource for investigating the high-temperature tolerance of this species and exploring the genomic basis that underlies the host range evolution of spider mites.

## Background & Summary

Phytophagous spider mites in Tetranychidae comprise more than 1,300 species, many of which are serious agricultural pests, such as *Tetranychus urticae* Koch, *Panonychus citri* McGregor^1^. Spider mites are notorious for developing rapid resistance to pesticides, causing significant economic losses since the widespread use of synthetic insecticides and fungicides after World War II^2^. The global acaricide market was valued at up to 400 million dollars annually^3^. In addition to their economic importance, spider mites exhibit diverse host ranges, from monophagous (e.g. *Tetranychus lintearius*) to extremely polyphagous (e.g. *T. urticae*)^4^, making them an ideal system for exploring the mechanisms underlying the evolution of host range. The two-spotted spider mite (*T. urticae*) is the first species of Chelicerate to have its whole genome sequenced, which was obtained by Sanger sequencing and assembled at the scaffold level^5^. Subsequently, in 2019, the genome was further refined and anchored into three pseudochromosomes^6^. This genome provides important insights into the host adaptation and pesticide resistance evolution of *T. urticae* and suggests a possible link between its rapid development of pesticide resistance and its strong adaptive ability to host plants^5,7^.

*Tetranychus piercei* (McGregor) is a major pest on banana (*Musa* spp.), papaya (*Carica papaya*) and other crops in East Asia^8–10^. It can also feed on plants such as mulberry (*Morus alba*), rose (*Rosa sp*.), peach (*Prunus persica*), sweetsop (*Annona squamosa*), cassava (*Manihot esculenta*) and fig (*Ficus carica*)^4^. Being the phylogenetically older sister species of *T. urticae*, *T. piercei* has a narrower host range and distinct host plant preferences^11,12^. Notably, *T. piercei* exhibits greater tolerance to high temperature compared to *T. urticae*, positioning it as a potential replacement for *T. urticae* as a major pest in the context of global warming^13^. However, limited information on its genetic resources hinders our understanding of its strong tolerance to high temperature and the evolution of the detoxification system in Tetranychinae.

In this study, we employed a combination of PacBio continuous long reads, accurate Illumina short reads and chromosomal conformation capture (Hi-C) data to assemble a chromosomal-level genome of *T. piercei*, which includes 3 chromosomes and 11,881 protein-coding genes. Synteny analysis revealed dramatic chromosomal rearrangement between *T. piercei* and *T. urticae*. This high-quality genome will facilitate in-depth biological studies of *T. piercei* and enable exploration of the genomic basis underlying the host range evolution of spider mites.

## Methods

### Raw material collection

At least 100 wild spider mites, including larvae, nymphs, and adults, were collected from *Trachycarpus fortunei* in Sanya Hainan province (18.29°N, 109.47°E), a tropical region in China. We amplified the nuclear ribosomal internal transcribed spacer (ITS) sequence to confirm the species identification of *T. piercei*^14^. By backcrossing male sons with a virgin female, an isofemale line was constructed to minimize heterozygosity. The isofemale line was reared on potted soybeans at a population size of thousands for at least 20 generations.

### DNA and RNA sequencing

Total genomic DNA was extracted from more than 5,000 adult females using MagAttract® HMW DNA Kit. The PacBio 30 kb SMRTbell library was prepared with more than 5 μg gDNA using the SMRTbell^TM^ Prep Kit 2.0 (Pacific Biosciences). The mode of Continuous Long Read (CLR) was run on the Sequel IIe system, and generated 6.44 Gbp raw data (75-fold depth). Illumina whole-genome sequencing was prepared using a 350 bp-insert fragment library (150 bp paired-end) by Truseq DNA PCR-free Kit, which was further sequenced on an Illumina NovaSeq 6000 platform. High-throughput chromosome conformation capture (Hi-C) included cross-linking, HindIII restriction enzyme digestion, end repair, DNA cyclization, purification and capture. The Hi-C library with 300–700 bp insert size library was sequenced on the NovaSeq 6000 platform, and 8.45 Gbp reads were generated to scaffold chromosomes. Total RNA was extracted from 100 adult females feeding on common beans using TRIzol Reagent (Invitrogen, USA) according to the manufacturer’s instructions. RNA library was constructed using the VAHTS mRNA-seq v2 Library Prep Kit (Vazyme, Nanjing, China) and sequenced on an Illumina NovaSeq 6000 platform. Finally, we generated 6.44 Gb (75×) PacBio long reads, 13.10 Gb (152×) Illumina short reads, 8.45 Gb Hi-C (98×) reads, and 12.62 Gb transcriptome reads for our genome assembly.

### Genome survey

**Fig. 1:**
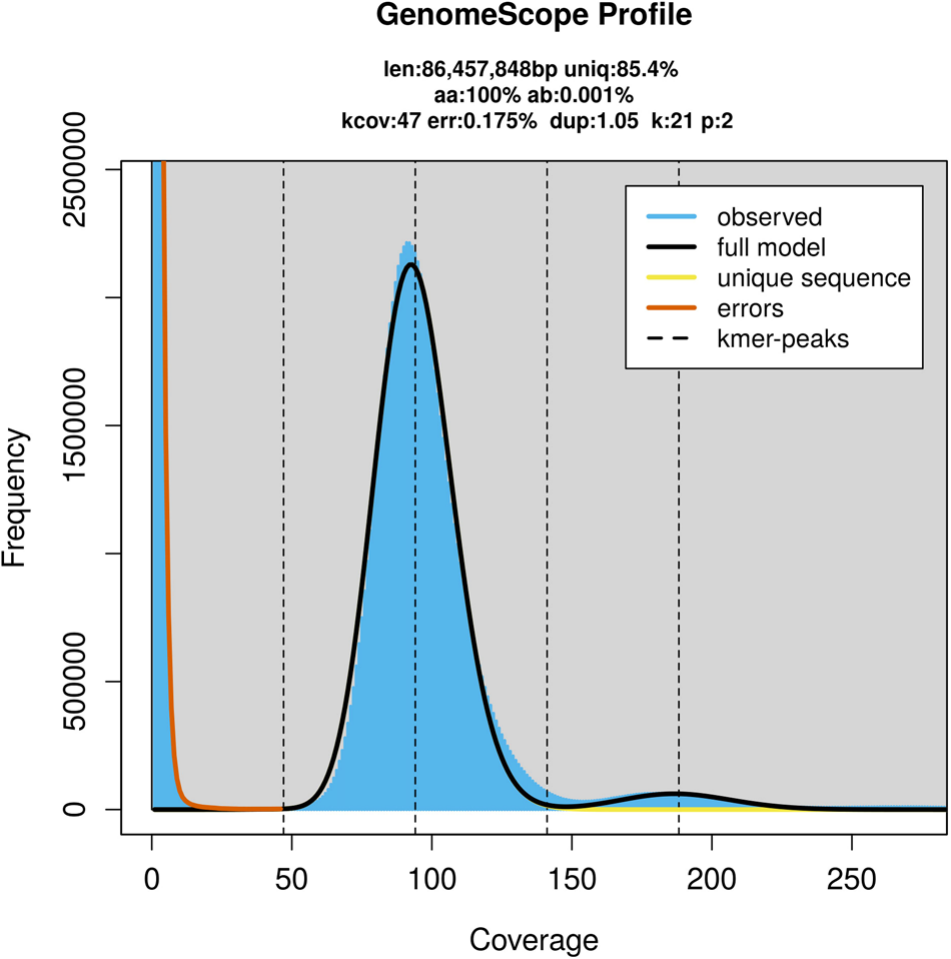
Genome survey at 21-mer of *T. piercei* estimated by GenomeScope. The vertical dotted lines represent the peaks of different coverages for the heterozygous, the homozygous, and the duplicated sequences (the last two peaks) separately.

Duplicate and low-quality Illumina raw reads (base quality < Q20, length < 15 bp, polymer A/G/C > 10 bp) were trimmed and removed using BBtools package v38.82^15^. The 21-mer depth distribution was counted using script khist.sh of BBtools v38.82. GenomeScope v2.0^16^ was used to estimate the genome size and heterozygosity of *T. piercei* with the maximum kmer coverage at 1,000×. Based on the distribution of kmer coverage and frequency, the estimated genome size of *T. piercei* was 86.45 Mb, with a heterozygosity rate of around 0.001% and a repeat content proportion of approximately 14.6% (Fig. 1).

### Chromosome staining

**Fig. 2:**
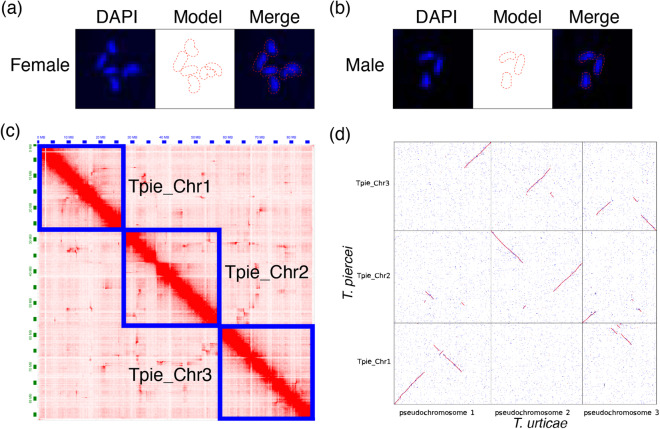
Chromosome staining, Hi-C heatmap and synteny. (**a**) DAPI staining of chromosomes in diploid female egg of *T. piercei*. The blue signals represent condensed chromosomal regions, while the red dashed lines in the model panel represent simulated chromosome boundaries. (**b**) DAPI staining of chromosomes in haploid male egg. (**c**) Genome-wide chromosomal interaction heatmap generated in Hi-C interaction analysis with each chromosome in the blue box. The frequency of Hi-C interaction links is represented by the color, which ranges from white (low) to red (high). (**d**) Synteny dot plot based on protein homologous between *T. piercei* and *T. urticae*.

Unfertilized (haploid) and fertilized (diploid) eggs of *T. piercei*, laid within 8 hours, were collected in a centrifuge tube containing phosphate buffered saline (PBS; 0.85% NaCl, 1.4 mM KH_2_PO_4_, 8 mM Na_2_HPO_4_, PH 7.1). The PBS was then discarded, and 500 μL 50% sodium hypochlorite solution was added, allowing it to stand for 2–3 minutes. After discarding the sodium hypochlorite solution, a mixture of hexane and methanol (1:1) was added to the centrifuge tube, and the contents were vigorously shaken for 3 min to remove the chorion. The eggs were rehydrated through an ethanol series (95, 70, 50 and 35%) and then washed in PBT (PBS containing 0.1% Triton X-100) for 15 min. After being washed five times for 1 min each in PBS, the eggs were incubated with a fluorescence quenching agent containing DAPI at room temperature for 5 min. Subsequently, the eggs were mounted on slides and covered with coverslips for further microscopic investigation using a Leica TCS SP8 confocal microscope. The egg DNA staining of diploid females and haploid males (Fig. 2a,b) consistently indicates that the genome of *T. piercei* consists of three chromosomes.

### Genome assembly

The CLR reads were set as input to Flye v2.9 and Raven v1.6.0 to assemble continuous long reads^17,18^. The better assembly from Flye, which had a greater N50 length and completeness, was retained as the following primary assembly. One round of built-in long reads polishing was performed by Flye v2.9. Then, two rounds of short reads were used to polish and fill in gaps of the primary assembly with NextPolish v1.4.0^19^. Haplotigs and duplication caused by haplotype divergence were eliminated by Purge_dups v1.2.5 using the alignment program Minimap2 v2.23^20,21^. Hi-C reads were aligned to the purged genome using BWA v0.7.17^22^ to anchor, order and orient contigs into chromosomal assembly following 3D-DNA pipeline^23^. Then, we manually reviewed and corrected assembled errors using Juicebox v1.11.08^24^. Vector contaminants were checked against the UniVec database using BLAST + v2.11.0 with the VecScreen parameters^25^. Bacterial and human being contaminants were detected in the assembly against the Nt database using MMseqs v13-45111^26^. The completeness of genome assembly was evaluated by BUSCO version 5.2.2^27^ using the arachnida_odb10 dataset (creation date 2020-08-05). The reads from the whole genome sequencing were aligned back to the genome assembly to access the mapping rate.

**Fig. 3:**
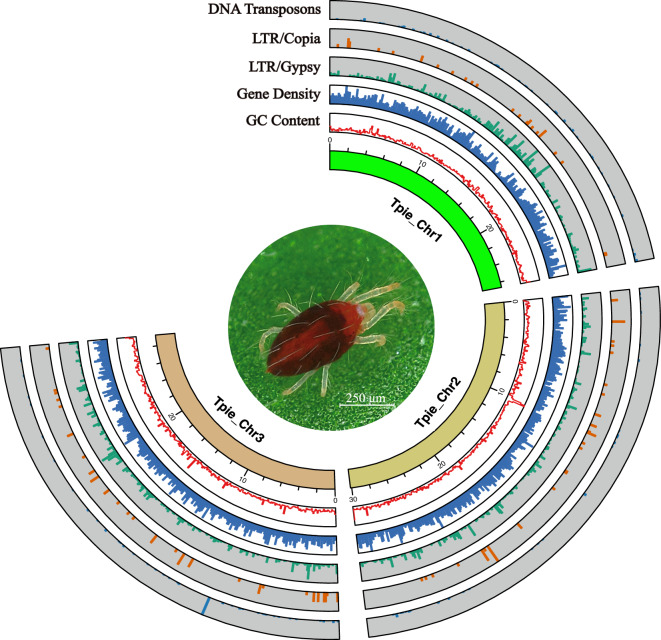
Circular karyotype representation of the chromosomes in non-overlapping windows of 100 kb. Tracks from inside to outside are GC content, gene density, Gypsy density, Copia density and DNA transposons density.

After *de novo* assembly, polishing and purging, 56 contigs (N50 of 3.33 Mb) with a total length of 86.13 Mb were generated, accounting for 99.63% of the estimated genome. By combining Hi-C with high-throughput sequencing and manual adjustment, we anchored these contigs into 4 scaffolds, including 3 megascaffolds and 1 unplaced scaffold (Fig. 2c). This unplaced scaffold was identified as bacterial contamination by NCBI and was subsequently excluded. Finally, a total length of 86.02 Mb contigs was assigned to 3 pseudochromosomes (Fig. 3), with scaffold N50 of 29.25 Mb (Table 1). The GC content of the *T. piercei* genome was 32.17%, which is similar to that of *T. urticae* (32.25%).

**Table 1:** Statistics for the assembly and annotation of *Tetranychus* genome.

Metric		*Tetranychus piercei*	*Tetranychus urticae*
Assembly	Length of contigs	86.13 Mb	90.83 Mb
	Length of chromosome	86.02 Mb	85.75 Mb
	Contig N50	3.33 Mb	0.21 Mb
	Scaffold N50	29.25 Mb	29.22 Mb
	Contigs number	56	1996
	Scaffolds number	4	601
	Pseudochromosomes number	3	3
	Length of Chr1	26,633,653 bp	32,654,540 bp
	Length of Chr2	30,138,663 bp	29,215,314 bp
	Length of Chr3	29,245,017 bp	23,884,949 bp
	GC%	32.17	32.25
	BUSCO completeness	94.6%	94.6%
Annotation	No. of protein genes	11,881	19,102
	Mean protein length	494.7 aa	363 aa
	Exons per gene	3.35	3.53
	Mean exon length	459.6 bp	362.5 bp
	Introns per gene	2.46	2.87
	Mean intron length	603.0 bp	501.7 bp
	BUSCO completeness	94.5%	90.4%

### Genome annotation

The repetitive elements were identified using RepeatModeler v2.0.2, which discovered the complete long terminal repeats (LTR) with the integration of LTRharvest and LTR_retriever^28^. RepeatMasker v4.1.2p1 and RMBlast v2.11.0 were searched against the custom repeat library of Dfam 3.5 and Repbase v20181026 to soft mask repeats of the genome assembly^29–31^. Two *ab initio* gene prediction software, BRAKER2 v2.1.6 and GeMoMa v1.8^32,33^, were used to find protein-coding gene structure based on the masked genome. Transcriptome mapping conducted by HISAT2 v2.2.0 and homologous proteins from five species (*Daphnia magna*, *Dermacentor silvarum*, *Drosophila melanogaster*, *T. urticae*, and *Varroa destructor*) were provided to assist gene prediction of BRAKER2/GeMoMa. Genome-guided transcript assembly was performed by StringTie v2.1.6, and the results were used as mRNA evidence for MAKER2 v3.01.03^34,35^. The same homologous proteins were also fed to MAKER2 as the protein evidence. Finally, MAKER2 combined *ab initio* prediction, mRNA and homology-protein evidence to generate gene models with direct predictions not allowed for transcripts and proteins. Proteins with lengths shorter than 30 aa were discarded. The functional annotation (Table S1) of predicted protein sequences was searched against UniProt, InterProScan and eggNOG databases. Diamond v2.0.11^36^ under the ‘very sensitive’ mode was used to assign gene function of the best hits in the UniProt database. Protein domains, Gene Ontology terms and pathways were supported by Pfam, SMART, Superfamily and CDD using InterProScan5^37^. Complementary annotation of ko, KEGG categories were provided using eggNOG-mapper v2.1.5 against EggNOG 5.0 database^38,39^.

To explore chromosomal synteny between *T. piercer* and *T. uritcae*^6^, we conducted a homologous search between their respective protein sequences using Diamond v2.0.11^36^ with default parameters. The resulting homologous dot plot was visualized to detect collinearity, inversions and translocations using WGDI v0.6.5^40^.

## Data Records

The raw reads and genome assembly have been deposited in the NCBI databases under BioProject PRJNA833563. The PacBio, Illumina, Hi-C, and transcriptome data are available under identification numbers SRR23622209-SRR23622212^41^. The final chromosome assembly has been deposited at GenBank under the accession number GCA_036759885.1^42^. The final chromosome-level genome assembly, annotation, and protein sequences are available at the Figshare database (10.6084/m9.figshare.22215145)^43^.

## Technical Validation

### Evaluation of the genome assembly

Compared to *T. urticae*^6^, our genome assembly exhibits greater contiguity and completeness attributed to the utilization of long reads and Hi-C sequencing. The contigs number, contigs N50 of *T. piercei* are much better than those of the two-spotted spider mite (56 vs. 1,996; 3.33 Mb vs. 0.21 Mb; Table 1). We mapped whole-genome resequencing reads to the *T. piercei* genome and found that 92.5% of PacBio long reads and 96.51% of Illumina short reads could be well aligned. The complete benchmarking universal single-copy orthologs (BUSCOs) under genome mode were used to assess the genome completeness. A total of 94.6% (2,775/2,934) complete BUSCOs were identified, including 89.0% (2,612) single-copy BUSCOs, 5.6% (163) duplicated BUSCOs, 0.7% (22) fragmented BUSCOs, and 4.7% (137) missing BUSCOs.

### Repeat elements and protein-coding genes

A total of 12.20 Mb repetitive elements were identified, accounting for 14.16% of the genome (Fig. 3). Besides unclassified repeats (4.67%), long-terminal repeat (LTR) represented the most common repeat element (4.36%), followed by simple repeats (2.04%), DNA transposons (1.67%), long-interspersed elements (LINE, 0.68%), and others (0.75%).

Using multiple lines of evidence, we annotated 11,881 protein-coding genes for *T. piercei* (Table 1). Most genes (>97%) had ‘annotation edit distance’ (AED) scores smaller than 0.5, indicating strong support from the evidence of transcript and homologous protein. Under the protein model, the complete BUSCOs for our genome annotation were 2,773 (94.5%). Although *T. urticae* had more coding genes than *T. piercei* (19,102 vs. 11,881), the results of BUSCOs suggested that *T. piercei* had higher completeness (90.4% vs. 94.5%; Table 1). Based on the synteny of homologous proteins, we found that the chromosomes of the two species underwent dramatic inversion and translocation events (Fig. 2d). More than half of Chr3 in *T. piercer* is syntenic to fragments of Chr1 and Chr2 in *T. urticae*.

### Supplementary information

Supplementary table

## Data Availability

All commands and pipelines were executed following the manuals and protocols of the corresponding bioinformatic software. The versions and parameters of the software have been detailed in the Methods section.
